# The immiscibility of InAlN ternary alloy

**DOI:** 10.1038/srep26600

**Published:** 2016-05-25

**Authors:** Guijuan Zhao, Xiaoqing Xu, Huijie Li, Hongyuan Wei, Dongyue Han, Zesheng Ji, Yulin Meng, Lianshan Wang, Shaoyan Yang

**Affiliations:** 1 Key Laboratory of Semiconductor Materials Science, and Beijing Key Laboratory of Low Dimensional Semiconductor Materials and Devices, Institute of Semiconductors, Chinese Academy of Sciences, P.O. Box 912, Beijing 100083, People’s Republic of China

## Abstract

We have used two models based on the valence force field and the regular solution model to study the immiscibility of InAlN ternary alloy, and have got the spinodal and binodal curves of InAlN. Analyzing the spinodal decomposition curves, we obtain the appropriate concentration region for the epitaxial growth of the InN-AlN pseudobinary alloy. At a temperature most common for the epitaxial growth of InAlN (1000 K), the solubility of InN is about 10%. Then we introduce the mismatch strain item into the Gibbs free energy, and the effect of different substrates is taken into consideration. Considering Si, Al_2_O_3_, InN, GaN, AlN as a substrate respectively, it is found that all the five systems are stabilized with the upper critical solution temperature largely reduced. Finally, InN and GaN are potential substrates for In-rich InAlN, while AlN and GaN substrates are recommended in the Al-rich region. Si and Al_2_O_3_ may be ideal substrates for thin InAlN film.

Nowadays, the group III nitrides are being actively investigated for their promising potentialities in optoelectronic devices of the visible/ultraviolet spectral range^1^,^2^,^3^. InN, AlN and their solution are a little new compared with other group III nitrides, but are now attracting much attention as potential materials for light emitting diodes (LEDs) and laser diodes (LDs)^4^,^5^. InN and AlN are direct bandgap semiconductors, and the bandgap of InAlN varies from 0.7 eV~6.2 eV with In composition continuously^6^,^7^,^8^. However, the large lattice mismatch between the InN lattice and the AlN lattice, which is of the order of 13%, causes difficulties in the growth of this alloy. Therefore, the prediction of miscibility gap in InAlN ternary compound becomes the subject of studies, and some groups have done research on it^9^,^10^,^11^,^12^,^13^,^14^,^15^.

Conventionally, the thermodynamics of multi-component nitrides is considered within the regular solution approximation, assuming the enthalpy of mixing to be controlled by the only interaction parameter *Ω* independent of temperature and alloy composition^9^. A lot of studies have been done to evaluate the interaction parameter, and several models have been set up. The delta-lattice parameter (DLP) model^16^ by Stringfellow, the valence force field(VFF) model^17^ by Keating, the Statistical model^9^ by Karpov *et al*., and the Molecular simulation method^10^,^11^ introduced into the examination of the phase and structural properties by Adhikari *et al*. are representatives of the models. The VFF model is an effective approach to obtain the interaction parameter theoretically without any adjustable parameters^18^. On the other side, Burton *et al*.^19^ and Liu *et al*.^20^ used the first principles calculations and cluster expansion to describe the energy functional and employed the Monte Carlo simulations to obtain the phase diagram.

In this paper, we not only use the regular solution model approximation to consider the thermodynamic properties of InAlN ternary alloy and two models based on the valence force field (VFF) to evaluate the interaction parameter, but also introduce the mismatch strain item into the Gibbs free energy and consider the effect of different substrates. Considering Si, Al_2_O_3_, InN, GaN, AlN as a substrate respectively, it is found that all the five systems are stabilized with the upper critical solution temperature (UCST) largely reduced.

## The Model

### Regular-solution theory

Regular-solution theory is an approximation of the actual system, and is proved to provide a good description of the thermodynamic properties of the alloys^10^. The central approximation of regular-solution theory is the neglect of the excess entropy and volume of mixing^21^, derived from the assumption of random mixing. Therefore, the excess Gibbs free energy is equal to the excess enthalpy. The molar free energy of mixing for InAlN can be approximated as^22^


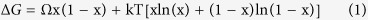


where *Ω* is the regular solution model interaction parameter, *x* is Indium composition*, k* is the Boltzman constant and *T* is the thermodynamic temperature. The first term on the right hand side of Eq. (1) is the enthalpy of mixing given by the regular solution theory, i.e.,


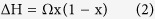


the second term in Eq. (1) is the ideal configurational entropy of mixing based on the assumption of a random distribution of In and Al atoms. The equilibrium solubility of InN and AlN (the binodal point at a given temperature) is calculated as the composition where 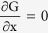 22. The spinodal composition is that where
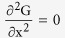
at a given temperature^22^. The interaction parameter, *Ω*, is the only parameter in Eq. (1) that must be determined for the InAlN system. Regular-solution theory provides a good description of the thermodynamic properties of the alloys, and comparison of the simulation results with the phase behavior previously reported using regular-solution theory finds good agreement^10^.

### Two models based on the valence force field

To study the effect of boundaries between the core atoms around an impurity and the rest of the elastic medium, various models based on the VFF are derived. In this section we consider two models based on the valence force field, and they are from Chen *et al*’.s article^23^. Of all the models listed there, we pick out Model A and Model D2, which involve more atoms shell in the calculation process and obtain more precise results. The two models produce the correct impurity bond lengths with variances for the compounds studied about equal to the experimental uncertainties in EXAFS, particularly on (In, Al) substitution system^23^. Using these two models, we determine the interaction parameter *Ω*, then we determine the phase diagram of InAlN.

## Model A: Third-shell atoms and beyond are fixed at their pure crystal positions

Consider the problem of substituting an isoelectronic atom A for a B atom in a zinc-blende compound BC (e.g., In substitutes for Al in AlN). The bond lengths surrounding the impurity A (first-shell) is *d*_1_ = *d* (1 − δ) and the second-shell atoms have radial displacements of the forms (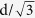) (*γ, γ*, 0), etc, where *d* is the equilibrium bond length of binary compound BC. Beyond and including the third shell, all the other atoms are held at their pure-crystal positions. There are nine contributions for Δ*E* of VFF: the bond stretching and bending of the first-shell bonds, the second shell bonds and the third shell bonds; the bond bending energy between the nearby shell bonds from the first-shell to the fourth-shell respectively. Thus, the excess energy (in this case 1/4 times the strain energy) becomes^23^


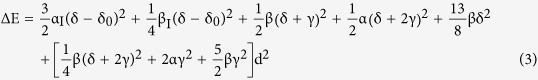


The minimization of Δ*E* with respect to *δ* and *γ* leads Eq. (3) to γ = −δ/4, and


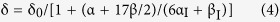


where *α, β* are the bond stretching and the bond bending force constants of the pure binary compound, respectively, *α*_*I*_, *β*_*I*_ are the bond stretching and the bond bending force constants of the “impurity” compound, respectively, and *δ*_*0*_ is the relative difference of bond length between the “impurity” compound and the pure binary compound.

## Model D2: VFF with the continuum connected to the second-shell atoms

In this case, Δ*E* only contains the first five contributions of the bond streching and bending force among the replacing atom, the first-shell atoms and second-shell atoms plus the elastic energy in the continuum term of other atoms (beyond and including the third-shell atoms)^23^.


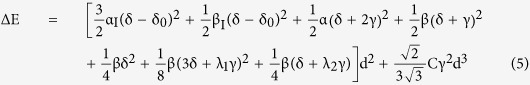


the corresponding *δ* becomes


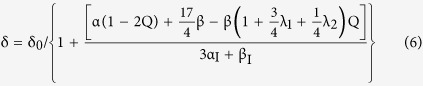


where


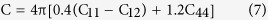



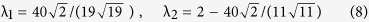



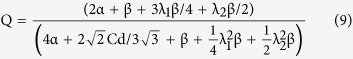


where C_11_, C_12_ and C_44_ are elastic parameters and the other parameters have been defined previously.

### The interaction parameter and corresponding parameters used in its determination

The interaction parameter *Ω* is given by^23^.


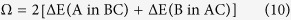


where A can be considered as In, and BC can be considered as AlN, with B being Al and AC being InN.

Table 1 lists lattice parameters of InN, AlN, GaN, Al_2_O_3_ and Si at the temperature of 300 K^24^,^25^. Table 2 lists the elastic parameters used in this paper^26^.

### Effect of mismatch strain

When the interface plain is perpendicular to the hexagonal axis of the wurtzite crystal, the elastic energy per mole is given by the expression^27^.


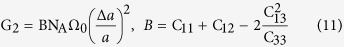


where *N*_*A*_ is Avogadro’s constant, Δ*a* = *a* − *a*_s_, *C*_*ij*_ (i, j = 1…6) are the elastic stiffnesses of the material and 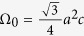 is the molecular volume of the ternary compound. The lattice constants of the ternary compound as well as the effective elastic constant *B* are assumed to obey the Vegard’s law


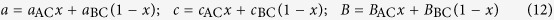


based on the parameters listed in Table 2 and equation (11), the effective elastic constant ***B*** can be calculated to 306.64 GPa for InN and 509.61 GPa for AlN.

## Results and Discussion

The interaction parameter *Ω* is determined to be 53.88 kJ/mol and 43.92 kJ/mol respectively, based on Model A and Model D2. We note that Model A produces *Ω* values about 20% larger than Model D2 because their way of estimating *Ω* is different. The VFF model is found to overestimate the total strain energy of the ternary system due to the constraint that only one of the two sublattices is allowed to relax. The values of *Ω* decrease significantly as the number of neighboring shells allowed to relax increases from Model A to Model D2^22^.

### Without the mismatch strain in consideration

Using the equations listed above, the phase diagram of InAlN with the spinodal curve can be given as


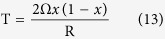


and the binodal curve is given as


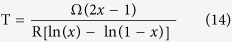


The phase behavior is summarized in an InN-AlN pseudobinary phase diagram, in which the upper critical temperature, the equilibrium composition and metastable composition can be obtained. In the region above *T*_*C*_, the solid solution is miscible in all proportions. Below *T*_*C*_, phase separation occurs between the two temperature-dependent metastable compositions, and there is a limit to the solubility of each constituent.

**Figure 1:**
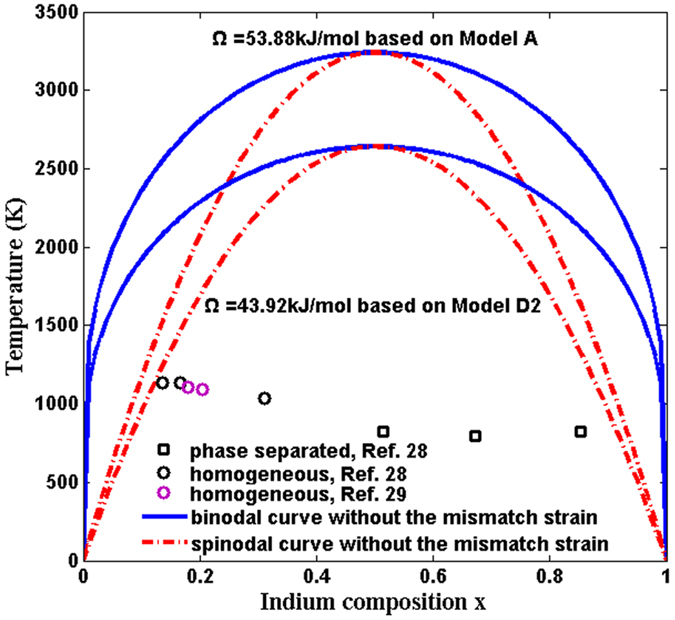
Binodal (continuous lines) and spinodal (dashed lines) curves for the InAlN system: *Ω* = 53.88 kJ/mol based on Model A, *Ω* = 43.92 kJ/mol based on Model D2. The data from refs 28 and 29 are plotted as circles for homogeneous and squares for phase separated InAlN, respectively.

Figure 1 is the phase diagrams based on the two models. It provides values for the upper critical solution temperature (UCST) of the InAlN ternary alloy: 3240 K and 2640 K respectively. The former is close to Takayama *et al*.’s result^12^, which is 3400 K, and the latter is equal to Adhikari *et al*.’s result^10^ and Burton *et al*’.s result^19^. As shown in Fig. 1, the equilibrium solubility of the two binary compounds in InAlN is nearly 0, and the maximal metastable solubility is around 10% at the temperature most common for the epitaxial growth of InAlN (1000 K^28^,^29^): it is a little less than 10% for Model A, and a little more than 10% for Model D2, actually. These results indicate that InAlN alloys are unstable over most of the composition range at normal growth temperatures without the mismatch strain.

### With the mismatch strain in consideration

**Figure 2:**
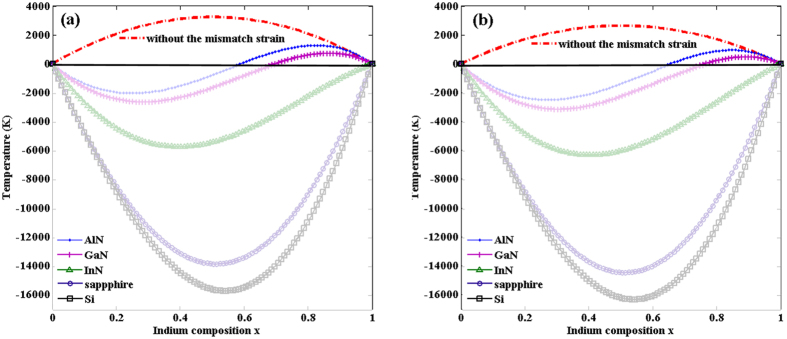
Spinodal curves of InAlN with different substrates, the substrates are signed on the figure: (**a**) *Ω* = 53.88 kJ/mol based on Model A, (**b**) *Ω* = 43.92 kJ/mol based on Model D2.

Introduce the mismatch strain energy into the Gibbs free energy of the InAlN system, the phase diagram of InAlN of the spinodal curve can be given as


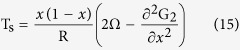


considering Si, Al_2_O_3_, InN, GaN, AlN as the substrates respectively, the *T-x* diagrams of InAlN with all are shown in Fig. 2 (Fig. 2a for model A and Fig. 2b for model D2). The critical temperatures are reduced as the coherency strain energy taken into consideration, which is consistent with Stringfellow’s result^16^. The *T*_*C*_ of the InAlN system with Si, Al_2_O_3_ and InN as a substrate are reduced significantly to negative temperature. For GaN or AlN as a substrate, the critical temperature of InAlN system is largely reduced in Al-rich region and small reduced in In-rich region compared with the fully relaxed situation (without the mismatch strain), which makes the T-x diagrams to wave shape. It’s not difficult to draw out the conclusion that biaxial strain increases the stability of the InAlN alloy^28^, and what’s more, in-plane tensile stress plays a more important role than in-plane compressive stress for the stabilization of the InAlN alloy. Deibuk and Voznyi supplementary take into account the biaxial strain of fully strained AlInN layers, which induces are distribution of the valence-electron charge density. As a result the composition in the strained layers is stabilized and the miscibility gap narrows^30^. As Si has the largest lattice parameter and InAlN is given the largest in-plane tensile stress in the InAlN/Si system, the InAlN/Si system is most stabilized with the lowest “*T* ” in the *T-x* diagrams. In the region rich in In, with AlN or GaN as a substrate, the system is close to InN/AlN or InN/GaN, in which InAlN is compressively stressed, therefore, the *T-x* diagrams have higher “*T* ” than the Al rich part.

**Figure 3:**
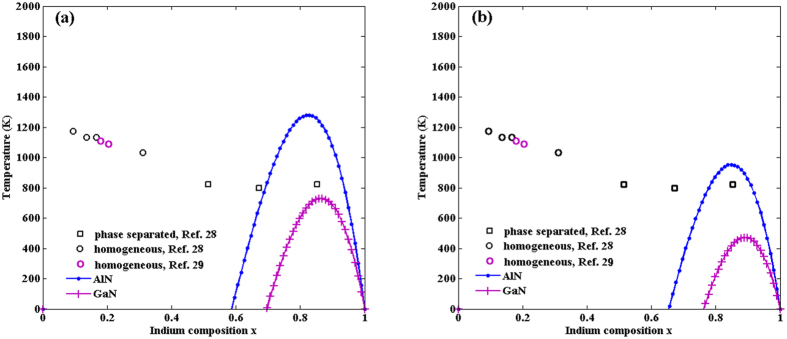
Spinodal curves of InAlN with GaN and AlN as a substrate, respectively: (**a**) *Ω* = 53.88 kJ/mol based on Model A, (**b**) *Ω* = 43.92 kJ/mol based on Model D2. The data from refs 28 and 29 are plotted as circles for homogeneous and squares for phase separated InAlN, respectively.

Figure 3 shows the *T-x* curves with AlN or GaN as substrates in the positive temperature range. The miscibility gap shifts remarkably into the area of higher Indium concentration and becomes of asymmetrical shape. The UCST is about 1280 K and 730 K respectively, for the InAlN/AlN and InAlN/GaN systems in Model A, compared to about 953 K and 473 K, in Model D2. Whether phase separation occurs or not for an experimental data is related to both of the fully relaxed curve and the fully strained one. Our result corresponds to Hums *et al*.’s^28^ and Kret *et al*.’s^29^ experimental data. In the experiments, InAlN/GaN_(sub)_ samples with a growth temperature range of 760 °C–860 °C and an In content of 0.13 < *x* < 0.32 show no phase separation (the circles in Fig. 3), and beyond this range of In concentrations, phase separation is likely to occur (the squares in Fig. 3). The range of 0.13 < *x* < 0.32 is included in our region of miscibility (it is miscible in all the concentrations in this temperature range when the system is fully strained, actually). Although the InAlN/Al_2_O_3_ system is the most stable, it may induce cracks and dislocations in InAlN for the large lattice mismatch, so it is not recommended to use Al_2_O_3_ substrate directly if you intend to obtain thick films. As InN will be decomposed at high temperature, it is not suitable to use InN substrate in the region rich in Al as AlN has high growth temperature. And it may also be unadvised to use AlN substrate in the region rich in In, for the relatively higher *T*_*C*_ (1280 K for Model A and 953 K for Model D2), and it is likely to result in immiscibility especially when the system is not strained.

Above all, the conclusion is that InN and GaN are potential substrates for In-rich InAlN, while AlN and GaN substrates are recommended in the region rich in Al. Si and Al_2_O_3_ may be used when very thin InAlN films are grown.

## Conclusions

We have demonstrated theoretically that the miscibility gap exists in the InN-AlN pseudobinary phase diagram. The unstable two-phase regions are predicted from calculations based on the regular solution model, and the interaction parameters are obtained by two models based on the valence force field. As Model D2 results in lower interaction parameter and suggests smaller bond-stretching and bond-bending energy, it may be more close to the condition of a real system. So VFF with the continuum connected to the second-shell atoms are much more advised with a result of *Ω* = 43.92 kJ/mol and *T*_C_ = 2700 K. We have also introduced the mismatch strain item into the Gibbs free energy, and the effect of different substrates is taken into consideration. Considering Si, Al_2_O_3_, InN, GaN, AlN as a substrate respectively, it is found that all the four systems are stabilized with *T*_*C*_ largely reduced. Above all, we draw a conclusion that InN and GaN are potential substrates for InAlN rich in In, while AlN and GaN substrates are recommended in the region rich in Al. Si and Al_2_O_3_ may be ideal substrates for thin InAlN films.

## Additional Information

**How to cite this article**: Zhao, G. *et al*. The immiscibility of InAlN ternary alloy. *Sci. Rep.* **6**, 26600; doi: 10.1038/srep26600 (2016).

## Figures and Tables

**Table 1:** Lattice parameters of Si, Al_2_O_3_, InN, AlN, and GaN^24^.

	***a*****(Å)**	***c*****(Å)**
AlN	3.112	4.982
InN	3.545	5.703
GaN	3.189	5.185
Al_2_O_3_	4.758^25^	
Si	5.4308^25^	

**Table 2:** The elastic parameters of InN and AlN^26^.

	**α(N/m)**	**β(N/m)**	**β/α**	**C**_**11**_**(GPa)**	**C**_**12**_**(GPa)**	**C**_**44**_**(GPa)**	**C**_**33**_**(GPa)**	**C**_**13**_**(GPa)**
AlN	98.0	15.0	0.15	411	149	125	389	99
InN	79.2	7.1	0.09	271	124	46	200	94
